# Th17 Cells and IL-17 in Protective Immunity to Vaginal Candidiasis

**DOI:** 10.1371/journal.pone.0022770

**Published:** 2011-07-27

**Authors:** Donatella Pietrella, Anna Rachini, Mark Pines, Neelam Pandey, Paolo Mosci, Francesco Bistoni, Cristophe d'Enfert, Anna Vecchiarelli

**Affiliations:** 1 Microbiology Section, Department of Experimental Medicine and Biochemical Sciences, University of Perugia, Perugia, Italy; 2 Institute of Animal Science, Volcani Center, Bet Dagan, Israel; 3 Internal Medicine Section, Department of Veterinary Pathology, Diagnostic and Veterinary Clinic, University of Perugia, Perugia, Italy; 4 Institut Pasteur, Unité Biologie et Pathogénicité Fongiques, Paris, France; 5 INRA, USC2019, Paris, France; University of Minnesota, United States of America

## Abstract

**Background:**

Th17 cells play a major role in coordinating the host defence in oropharyngeal candidiasis. In this study we investigated the involvement of the Th17 response in an animal model of vulvovaginal candidiasis (VVC).

**Methods:**

To monitor the course of infection we exploited a new in vivo imaging technique.

**Results:**

i) The progression of VVC leads to a strong influx of neutrophils in the vagina soon after the challenge which persisted despite the resolution of infection; ii) IL-17, produced by vaginal cells, particularly CD4 T cells, was detected in the vaginal wash during the infection, reaching a maximum 14 days after the challenge; iii) The amount and kinetics of IL-23 in vaginal fluids were comparable to those in vaginal cells; iv) The inhibition of Th17 differentiation led to significant inhibition of IL-17 production with consequent exacerbation of infection; v) An increased production of βdefensin 2 was manifested in cells of infected mice. This production was strongly reduced when Th17 differentiation was inhibited and was increased by rIL-17 treatment.

**Conclusions:**

These results imply that IL-17 and Th17, along with innate antimicrobial factors, have a role in the immune response to vaginal candidiasis.

## Introduction

*Candida* vulvovaginal infection is a frequent and common distressing disease affecting 70%–75% of women of childbearing age worldwide at least once during their lifetime. 5%–10% of women with a primary episode of VVC subsequently experience frustrating recurrent infection (RVVC) [1], defined as at least three-four specific episodes within one year [2], [3]. There are two forms of RVVC: primary RVVC is idiopathic with unknown predisposing factors, secondary RVVC is the occurrence of frequent episodes of acute VVC because of certain predisposing factors such as hormone replacement therapy or diabetes mellitus [4].

It has been demonstrated that the vaginal mucosa, its tissue structure and cervicovaginal fluids, contains both humoral and cellular components of innate and acquired immune responses [5]. Animal models are frequently used to evaluate host defense mechanisms against *Candida* vaginitis [6]. *Candida albicans* is not a natural colonizer of the vaginal mucosa but the use of estrogen favors a persistent infection and this can be exploited in order to study the immune response [4], [5]. The mouse infection model has many advantages, such as its similarity with the human infection and the possibility of performing vaginal lavages to quantify the fungal burden. The major disadvantages are the requirement for a condition of pseudo-estrus induced by estrogen, the neutral vaginal pH, and the lack of symptoms [6].

The Th1 response has been shown to be induced in the systemic draining lymph nodes of vaginally infected mice, but does not protect against the infection [7]. Despite the evidence reported for competent vaginal cell-mediated immunity, resident T cells, during infection, appeared to have little or no protective role [8], [9], [10], [11], [12], [13].

Th17 cells belong to a lineage different from that of Th1 and Th2 cells, and they are characterized by the production of IL-17A, IL-17F and IL-22 [14]. The protective action of IL-17 against extracellular pathogens also involves neutrophil recruitment to the infection sites [15]. IL-17 has a central role in protective immunity against *C. albicans* systemic and oral infections [16], [17], [18]. In response to a systemic challenge with *C. albicans*, IL-17AR-deficient mice showed a reduced survival rate and a significant increase of kidney fungal burden. Mobilization and influx of neutrophils to infected organs were also impaired and delayed [16]. In another study, the Th17 response also conferred protection against oropharyngeal candidiasis through neutrophil recruitment and antimicrobial factor production [17]. In the present work, we focus on the role of IL-17 in protecting against vaginal candidiasis, exploiting a new in vivo imaging technique that we have recently developed [19], [20]. In the in vivo imaging method a *C. albicans* strain is used that expresses a cell surface luciferase constructed by fusing the *Gaussia princeps* luciferase to the *C. albicans* PGA59 glycosylphosphatidylinositol-linked cell wall protein [19]. This technique allows continuous, non-invasive monitoring of the spatial and temporal progression of vaginal infection in live mice and has proved useful to evaluate vaccinal and immunotherapeutic approaches to the treatment of vaginal candidiasis [20].

## Results

### Th17 response in murine vaginal infection by *Candida albicans*

**Figure 1:**
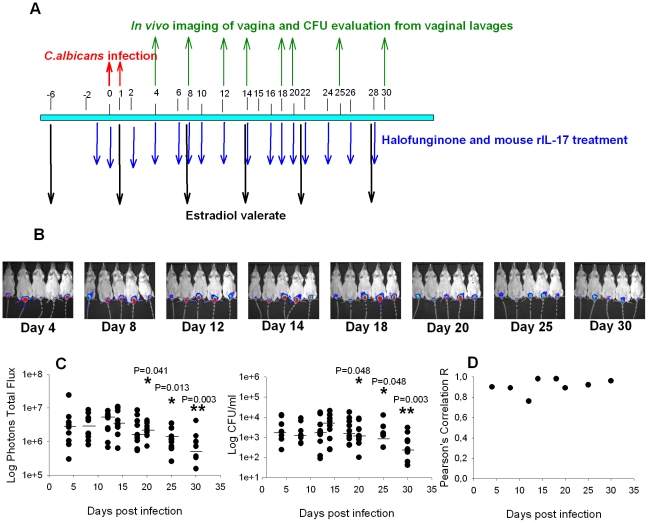
Model of murine vaginal infection and monitoring. (A) Timeline of vaginal infection model. CD1 mice are resistant to *Candida* vaginal infection unless they are treated with estradiol valerate. CD1 mice were treated subcutaneously with estradiol valerate and infected for two consecutive days with 10 µl of 10^9^/ml suspension of *Candida albicans* cells (gLUC) into vaginal lumen. Two days before and every two days after challenge mice were treated intraperitoneally with halofuginone or diluent of halofuginone and, in selected experiments, intravaginally with 10 pg of recombinant mouse IL-17. After 4, 8, 12, 14, 18, 20, 25, 30 days post infection, mice were treated intravaginally with 10 µg of coelenterazine and imaged in the IVIS-200™ imaging system under anaesthesia using 2.5% isoflurane and the vaginal lumen was washed with 150 µl of saline using mechanical pipette. The fungal burden of vaginal fluids was evaluated by colony forming units (CFU) assay. (B) In vivo imaging of mice vaginally infected with *Candida albicans* cells (gLUC). Images are representative of 5 out of 10 mice for each experiment. C) Dot plots of total photon emission from the infected vaginal regions and corresponding CFU in vaginal washes of infected mice (n = 10). The statistical analysis was performed by non-parametric Mann-Whitney U test. The median is indicated by a straight line. Data are representative of one of two independent experiments with similar results. D) The correlation between the Total Photons emitted and CFU count in the vaginal wash was assessed using the Pearson's correlation statistics, and the correlation coefficients are shown for each time point. * *p*<0.05, ** *p*<0.01, (Log Photons Total Flux or Log CFU/ml of mice infected after 8, 12,14,18,20,25,30 days vs Log Photons Total Flux or Log CFU/ml of mice infected after 4 days).

We investigated the role of Th17 during vaginal candidiasis using CD1 female mice maintained under pseudo-estrus conditions and vaginally infected with the pathogenic *C. albicans* gLUC59 strain as previously reported [19]. gLUC59 has been used to monitor the course of different types of *C. albicans* infection. Studies performed on mice infected with *C. albicans* strain CA1398 carrying the *ACT1p-gLUC* fusion (gLUC59), or the control strain CA1399 which did not express *gLUC59*, showed that these gLUC59 and control strains were equally pathogenic [19]. Data presented in Figure 1 show that significant luminescence signals, obtained following injection of the luciferase substrate coelenterazine in the vagina and animal imaging using the Xenogen IVIS-200™ imaging system, were evident after 4 days of infection and declined after day 18 post-infection (Fig. 1B). The analysis of the total photon emission showed a significant reduction of the fungal load from day 20 post-infection; a similar trend was observed by CFU counting from vaginal lavages from the same mice (Fig. 1C). Analysis of the two parameters measured to estimate the fungal burden, photon emission and CFUs, showed a good correlation between the results obtained with the two methods, for all experimental time points (Fig. 1D)

**Figure 2:**
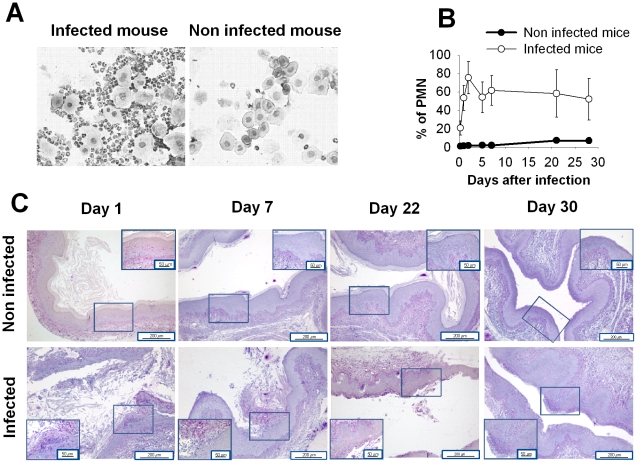
Polymorphonuclear cell influx in murine vaginal compartment of mice infected with *Candida albicans*. (A) Vaginal fluids obtained from *Candida* infected (left) and non infected (right) mice at day 2 post-infection were cytospun onto glass slides, air dried, stained using Diff Quick coloration, and analyzed by light microscope (magnification, ×40). (B) Percentage of polymorphonuclear cells present in vaginal washes of *Candida* infected mice and non infected mice at different times after infection. The cells present in vaginal lavages obtained by centrifugation of the total vaginal wash were stained with a polymorphonuclear cell antibody (anti mouse Ly-6G-FITC) and analyzed by FACS machine. Results are expressed as mean±SD (n = 12 mice, 4 mice for each of three separate experiments). (C) Histological evaluation of vaginal *Candida albicans* infection. At day 1,7, 22 and 30 after infection, five sections of vagina per mouse from two mice per group (above, non infected mice; below, infected mice) were stained with period acid-Schiff reagent and viewed (magnification, ×10 and ×40; bars 50 µm and 200 µm respectively). The pictures are representative of one of two independent experiments with similar results.

In selected experiments, a histological analysis of the vagina was performed and the cellular composition of the vaginal fluid was examined. Results reported in Fig. 2A show that different cell types were present in the vaginal fluid of infected mice, especially neutrophils and epithelial cells. In uninfected mice, mainly epithelial and, rarely, immune cells, were detected. The trend of the cellular influx in the vagina of infected mice was evaluated by cytofluorimetric analysis (Fig. 2B) showing that, 48 h post-infection, a massive infiltration of neutrophils was evident (75% of total cells). Subsequently, the level of neutrophils decreased to about 60% of the total cells, a level that was maintained until the monitoring of the *Candida* infection finished (Fig. 2B). The histological analysis performed on day 1 post-infection evidenced that intraepithelial microabscesses consisting of polymorphonuclear cells were only present in the vagina of infected mice (Fig. 2C). Moreover a massive presence of fungal hyphae with rare blastospores was observed in the vaginal lumen and in the superficial epithelial layer until day 22 post-infection (Figure 2C).

**Figure 3:**
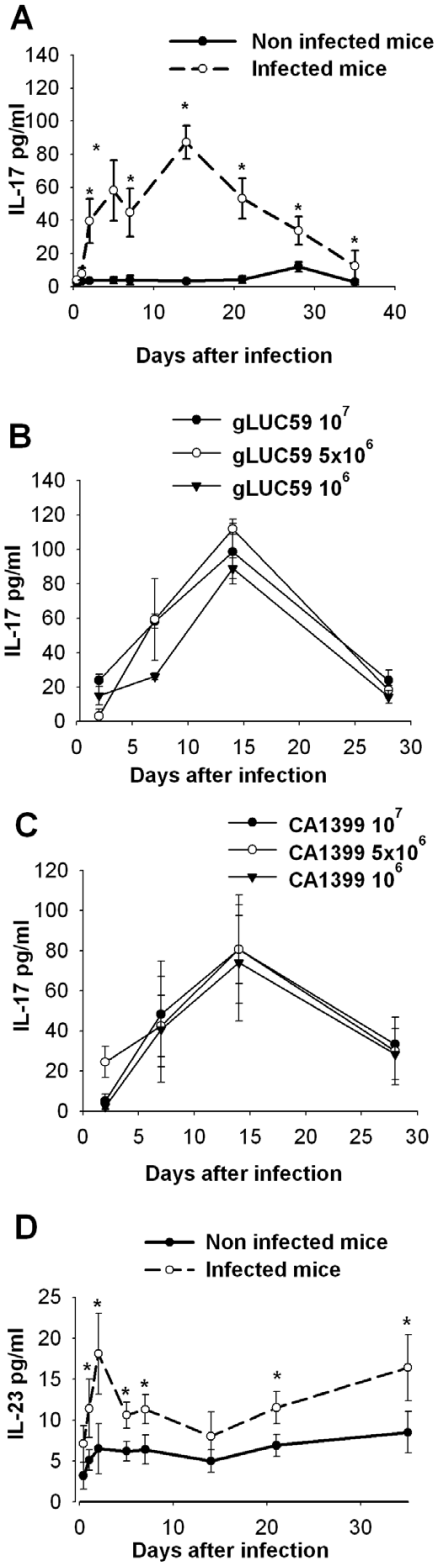
IL-17 and IL-23 concentration in murine vaginal washes of mice infected with *Candida albicans*. Evaluation of IL-17 (A–C) and IL-23 (D) concentration by ELISA test on supernatants of vaginal fluids obtained at different times after vaginal infection with different doses of *Candida albicans* gLUC59 (A–B) or CA1399 (D). Results are expressed as mean±SD (n = 12 mice, 4 mice for each of three separate experiments). * *p*<0.05, (infected mice vs non infected mice).

Because it has been demonstrated that IL-17 has a critical role in protecting against mucosal, particularly oropharyngeal, candidiasis [17], we investigated the presence of this cytokine in our model of vaginal infection. Results reported in Figure 3A demonstrate that there is an early production of IL-17, starting 48 h after the challenge, reaching a maximum 14 days post-infection, and subsequently decreasing to return to basal levels after 5 weeks of infection. The early production of IL-17 in the vaginal wash could presumably be attributed to PMN and epithelial cells, which are known to be innate system cells capable of producing IL-17 [21]. We also performed experiments using lower doses of *C. albicans*. The results showed that inocula of 10^7^, 5×10^6^ or 10^6^ are also able to induce IL-17 production (Fig. 3B). In a parallel experiment we evaluated the ability of the control strain of *C. albicans* CA1399 to induce IL-17 production. All concentrations of inocula used in the experimental vaginal infection induced cytokine production at day 14 (Figure 3C), as did the gLUC59 strain. It has been reported that elicitation of the IL-17 response correlated with the ability of *C. albicans* hyphae to stimulate antigen-presenting cells for the priming of Th-17 responses in vitro and for the production of IL-23, but not IL-12 [22]. IL-23 induces the differentiation of naive CD4(+) T cells into helper T cells that produce IL-17 [23]. Moreover, IL-23 appears to induce IL-17, IL-1 and IL-6 production from cells of the innate immune system [24]. Given this premise, we analyzed IL-23 levels in the intravaginal lumen. Results reported in Figure 3D show the presence of IL-23 in the vaginal wash of infected mice. An increased production of this cytokine was manifested 24 h after the challenge, reaching a maximum at 48 h, declining from day 3–10 post-infection and then increasing until the end of the monitoring. Importantly, IL-23 was recovered in vaginal fluids of infected mice at all time points except day 14 post-infection (Fig. 3D).

### Role of vaginal and lymph node cells in Th-17 response

**Figure 4:**
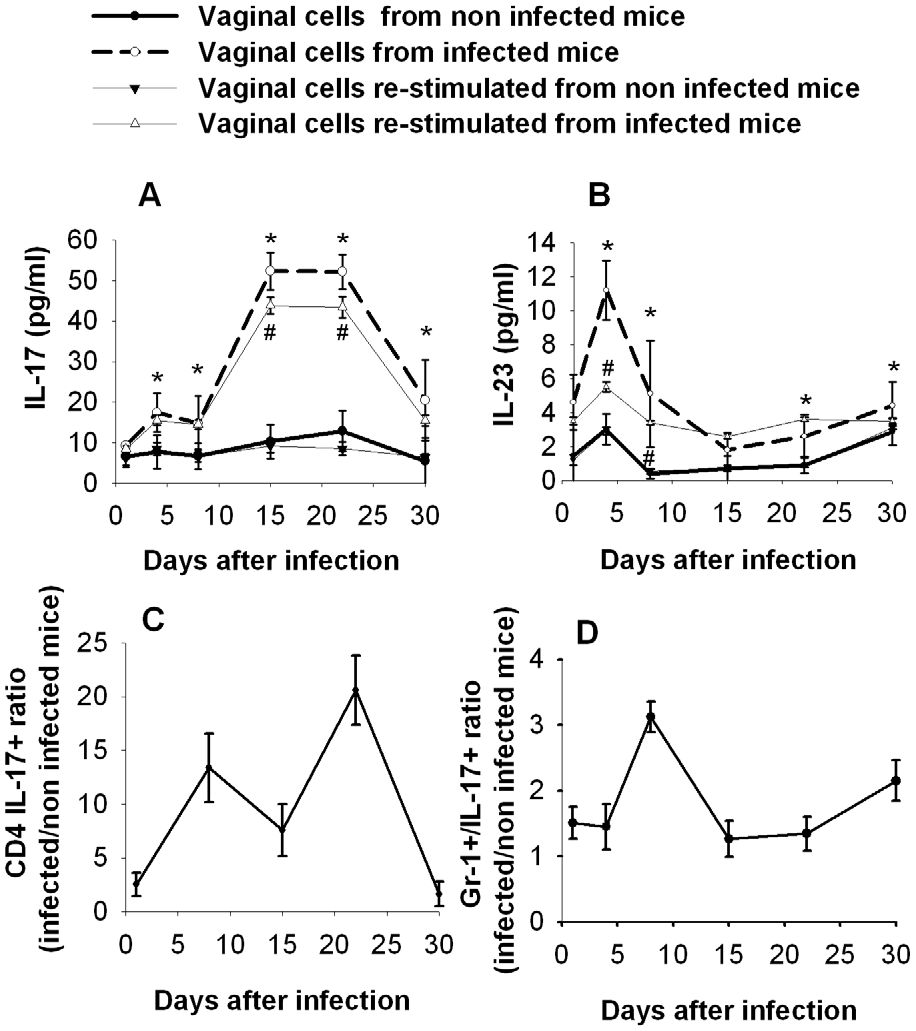
Effect of *Candida albicans* infection on vaginal cells. Vaginal cells were obtained at different times after vaginal *Candida* infection by collagenase digestion of whole vaginas aseptically recovered. Evaluation of IL-17 (A) and IL-23 (B) concentration by ELISA on supernatant fluids of 72 hour vaginal cells culture. Results are expressed as mean±SD (n = 16 mice, 4 mice for each of four separate experiments). The statistical analysis was performed by Mann-Whitney U test. * *p*<0.05 (cells from infected mice vs cells from non infected mice), # *p*<0.05 (vaginal cells re-stimulated from infected mice vs vaginal cells re-stimulated from non infected mice). Ratio between CD4-IL-17 positive cells (C) and between IL-17 positive Gr-1 positive cells (D) present in whole vagina of *Candida* infected mice and those present in the vaginas of non infected mice. The vaginal cells were stained with anti mouse CD4-FITC or a monoclonal antibody to mouse Gr-1 molecule expressed by neutrophils and with anti mouse IL-17-PE conjugate antibody for 45 minutes. After this they were analyzed by FACS machine. Results are expressed as mean±SD (n = 12 mice, 4 mice for each of three separate experiments).

In selected experiments the vagina and the lumbar lymph nodes from both infected and non infected mice were removed and vaginas were treated with collagenase. Results reported in Figure 4A demonstrate that IL-17 was produced by vaginal cells recovered from infected mice and that production reached a maximum 15–22 days post-infection, then declining. In a parallel analysis the production of IL-23 was tested in the same supernatants. Kinetic analysis showed a significant increase of IL-23 levels at day 4, which declined in the following days (Fig. 4B). A similar trend for IL-17 and IL-23 production was observed when vaginal cells were re-stimulated with heat-inactivated yeasts. To study the phenotype of cells producing IL-17, cells cultivated for 72 h were recovered, fixed, and labeled for Gr-1 or CD4 receptors. Intracellular IL-17 expression was evaluated in CD4 T cells and neutrophils, which are the major IL-17 producing cells. In our experimental conditions CD4 positive cells appeared the main source of IL-17, with maximum intracellular expression between days 8 and 21 (Fig. 4C). Yet some Gr-1 positive cells (neutrophils) also had appreciable levels of IL-17 intracellular expression seven days post infection (Fig. 4D).

**Figure 5:**
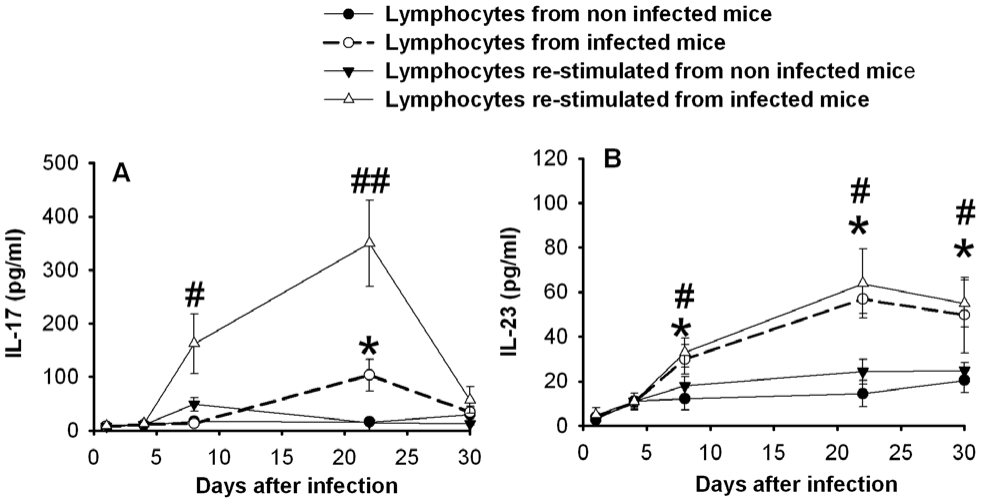
Effect of *Candida albicans* infection on draining lumbar lymph nodes. Lymph nodes, at different times after *Candida* infection, were aseptically recovered and mechanically homogenized. Cells were cultivated untreated or in presence of heat inactivated *C. albicans* for 72 hours. In the supernatant fluids of lymph node cell culture IL-17 (A) and IL-23 (B) were analyzed by ELISA. Results are expressed as mean±SD (n = 16 mice, 4 mice for each of four separate experiments). The statistical analysis was performed using Mann-Whitney U test. * *p*<0.05, (Lymphocytes from infected mice vs Lymphocytes from non infected mice). # *p*<0.05, ## *p*<0.01, (Lymphocytes re-stimulated from infected mice vs Lymphocytes re-stimulated from non infected mice).

Draining lymph nodes were also removed, cultivated for 72 h, then either left untreated, or stimulated with heat inactivated *C. albicans*, and the supernatant fluids were tested for the presence of IL-17 and IL-23. The results in Figure 5A show that IL-17 was produced by lymph nodes 22 days post-infection. After stimulation in vitro lymph node cells produced significant levels of IL-17 even at day 8, suggesting that some cells were already activated. A significant increase in IL-23 production was manifested 7 days after infection reaching a maximum at day 22 (Figure 5B). A similar trend was observed when cells were re-stimulated with heat inactivated *Candida* cells.

### Role of Th17 in vaginal *Candida* infection

**Figure 6:**
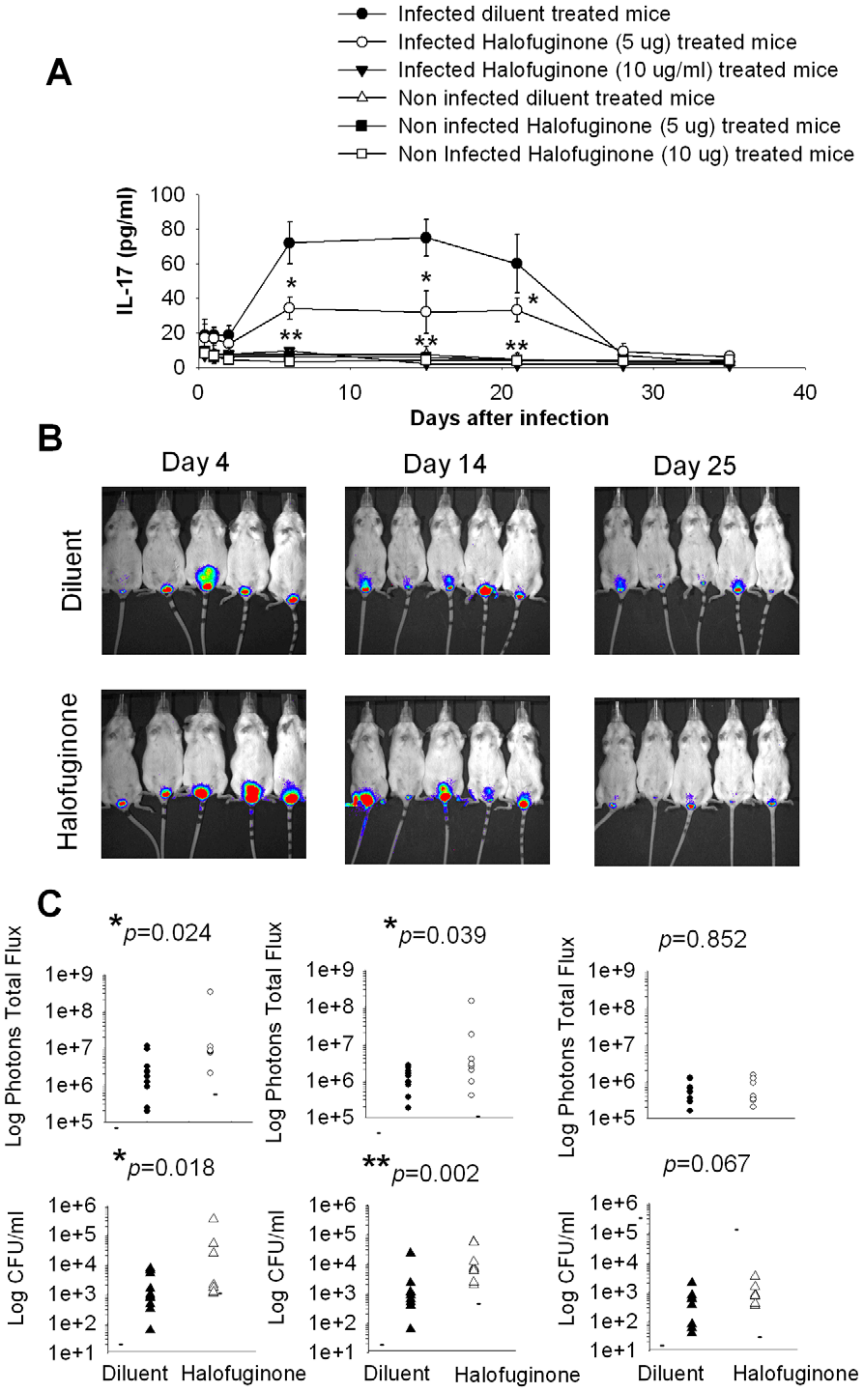
Effect of halofuginone on vaginal infection. Mice, under pseudo-estrus conditions, were twice infected with 10^7^ *Candida albicans* in vagina. Two days before and every two days after infection, mice were injected intraperitoneally with 5 µg/100 µl or 10 µg/100 µl of halofuginone solution or diluent of halofuginone and, in selected experiments, were treated intravaginally with 10 pg of mouse rIL-17. (A) Evaluation of IL-17 concentration by ELISA in supernatants of vaginal fluids obtained at different days after vaginal *Candida* infection and halofuginone treatment. Results are expressed as mean±SD (n = 9 mice, 3 mice for each of three separate experiments). The statistical analysis was performed using Mann-Whitney U test. * *p*<0.05, ** *p*<0.01 (infected halofuginone treated mice vs infected diluent treated mice). At day 4, 14 and 25 after infection, mice were treated intravaginally with 10 µg of coelenterazine and imaged in the IVIS-200™ imaging system under anesthesia using 2.5% isoflurane and the vaginal lumen was washed with 150 µl of saline. (B) In vivo imaging of mice vaginally infected with *Candida albicans* cells (gLUC) and treated with halofuginone or diluent. Images are representative of 5 out of 10 mice in two different experiments. (C) Dot plot of total photon emission from the infected regions and dot plot of CFU in vaginal washes of mice (n = 10) treated with halofuginone or diluent. The statistical analysis was performed using non-parametric Mann-Whitney U test. The median is indicated by a straight line. Data are representative of one out of two independent experiments with similar results. * *p*<0.05, ** *p*<0.01 (infected halofuginone treated mice vs infected diluent treated mice).

**Figure 7:**
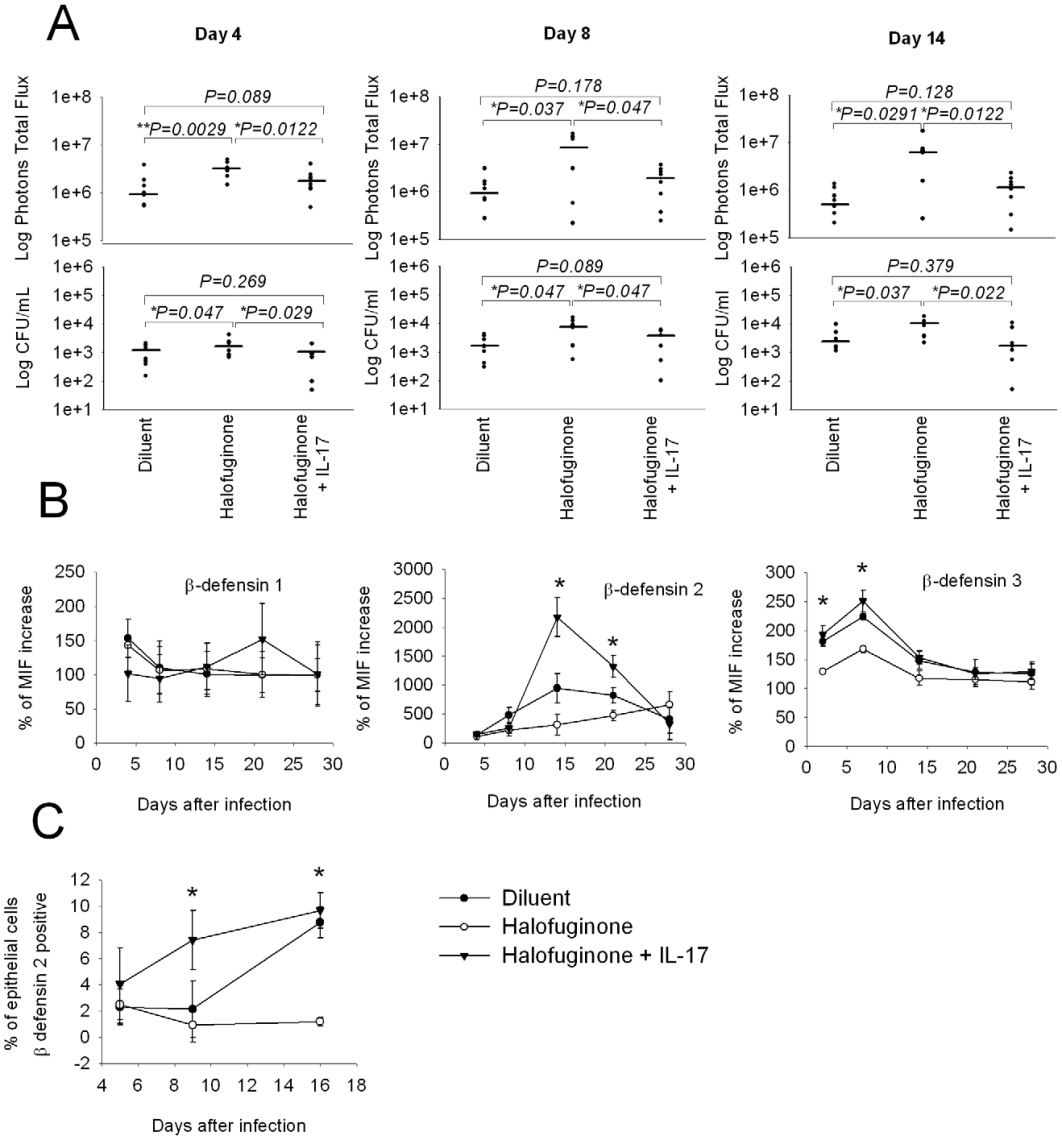
Effect of halofuginone treatment on vaginal β defensin production. Mice, under pseudo-estrus conditions, were twice infected with 10^7^ *Candida albicans* in vagina. Two days before and every two days after infection, mice were injected intraperitoneally with 5 µg/100 µl of halofuginone solution or diluent of halofuginone and were treated intravaginally with 10 pg of mouse rIL-17. At day 4, 8 and 14 after infection, mice were treated intravaginally with 10 µg of coelenterazine and imaged in the IVIS-200™ imaging system under anesthesia using 2.5% isoflurane. (A) Dot plot of total photon emission from the infected regions and dot plot of CFU in vaginal washes. The statistical analysis was performed using non-parametric Mann-Whitney U test. The median is indicated by a straight line. Data are representative of one out of two independent experiments with similar results. After 4, 8, 14, 21, 28 days from challenge, the vaginal lumen was washed with 150 µl of saline and vaginal cells were recovered for β-defensin analysis. (B) Mean of fluorescence MIF of β-defensin 1, β-defensin 2 and β-defensin 3 cells evaluated by FACS analysis. The vaginal cells recovered by vaginal washes were stained with rabbit anti-mouse BD1, goat anti-mouse BD2 or goat anti-mouse BD3 and goat anti-rabbit TRIC conjugate or rabbit anti-goat PE conjugate respectively. (C) Percentage of epithelial cells producing d β-defensin 2. Data are the mean±SD (n = 8 mice, 4 mice for each of two separate experiments). Cells of vaginal washes were stained with FITC anti mouse pan-cytokeratin and goat anti-mouse BD2 and rabbit anti-goat PE conjugate. Data are representative of one out of two independent experiments (total 8 mice). The statistical analysis was performed using Mann-Whitney U test. * *p*<0.05, (infected halofuginone+IL-17 treated mice vs infected halofuginone treated mice).

Recently it has been demonstrated that halofuginone is a specific potent inhibitor of mouse and human Th17 differentiation [25]. To analyze the role of Th17 response in our experimental model of vaginal candidiasis we treated mice with halofuginone every two days, starting two days before the infection (Fig. 1A). The analysis of IL-17 in the vaginal fluids recovered at different times after infection showed that the halofuginone treatment at a dose of 5 µg/mouse significantly lowered IL-17 production from day 6 to day 21. A dose of 10 µg/mouse completely suppressed IL-17 production (Fig. 6A). In addition, the fungal load evaluated from the bioluminescence intensity was found to be significantly higher in halofuginone treated mice (5 µg/mouse) with respect to diluent treated mice (Fig. 6B). This was correlated to a significant increase of CFU (507% fourteen days after infection and 208% twenty-five days after infection) in the vaginal wash of halofuginone treated mice (Fig. 6C). In further experiments, mice were treated with a higher dose of halofuginone (10 µg/mouse). In this case the increase of CFU was of 608% fourteen days after infection and 462% twenty-five days after infection. In parallel experiments the IL-17 depletion due to halofuginone treatment was restored by intravaginal administration of mouse recombinant IL-17 every 2 days. The fungal load was monitored 4, 8 and 14 days post infection. The bioluminescence and the CFU in rIL-17 treated mice were similar to those observed in the diluent treated mice used as a control group, and not statistically significant (Fig. 7A).

Antimicrobial peptides have an important role in the innate immune response of host cells [21] and are secreted particularly by vaginal epithelial cells [26], [27], [28]. We analyzed the kinetics of β-defensin 1, 2 and 3 (BD-1, BD-2 and BD-3, respectively) level in the vaginal wash at different days after infection. The results reported in Figure 7B demonstrate that halofuginone treatment produced a significant down-regulation of the production of BD-2 at day 14 and 21 (*p*<0.01). In contrast, intravaginal administration of rIL-17 together with halofuginone restored defensin production at day 14 and 21 (*p*<0.01). BD-1 was not produced. BD-3 was produced 2 and 7 days post infection, and halofuginone was also able to down regulate the production of this peptide. Moreover, the administration of recombinant IL-17 was able to restore the production of the antimicrobial factor. The cytofluorimetric analysis of cells recovered from the vaginal washes showed that the BD2 was produced by epithelial cells (Figure 7C). The increment of β-defensin 2 and 3 production after rIL-17 treatment of halofuginone treated mice correlated with a more rapid clearance of *C. albicans* (data not shown).

## Discussion

Mouse models of mucosal candidiasis, including gastrointestinal, oropharingeal and vaginal candidiasis, have provided an invaluable contribution to the understanding of the local immune response to *C. albicans*.

In this study we investigated Th17 responses during vaginal candidiasis and demonstrated that i) vaginal candidiasis leads to a strong influx of neutrophils to the vagina; 75% of the cells present in the vaginal wash soon after the challenge are neutrophils, a percentage which decreased to 60% until the resolution of infection; ii) IL-17 was detected during infection in vaginal fluids, its production reaching a maximum 14 days after the challenge and subsequently decreasing; iii) IL-17 was produced by CD4 T cells in particular, and there was a correlation between the presence of vaginal IL-17 and fungal burden; iv) an appreciable level of IL-23 was observed in the vagina and the amount and kinetics of IL-23 in vaginal fluids were comparable to those in vaginal cells; v) the inhibition of Th17 differentiation leads to significant inhibition of IL-17 production, with consequent exacerbation of infection; and vi) production of beta defensin 2 and beta defensin 3 was manifested in cells of infected mice, this production being strongly reduced when Th17 differentiation was inhibited and stimulated by rIL-17 treatment. Taken together, these results suggest that Th17 responses play a central role in protecting against vaginal candidiasis, possibly through their influence on antimicrobial peptide production by vaginal epithelial cells.

Here we demonstrate that the course and resolution of vaginal infection is strictly correlated to the presence of IL-17 secreted locally by CD4 vaginal T cells. This is consistent with a report showing that vaginal T cells have been characterized and quantified in the lamina propria and the epithelia of the vagina and cervix. It has been estimated that there are about 240 T lymphocytes per mm^2^ of vaginal epithelial tissue [29] and it is conceivable that the majority of vaginal T cells migrate to the vaginal epithelium in response to inflammatory chemokines following local antigenic stimulus. This increase of IL-17 in the vaginal wash mirrored the kinetics of IL-17 production by CD4 vaginal cells, however Gr-1 positive cells showed a marginal production of IL-17 too. It is conceivable that an early presence of IL-17 could be due to neutrophils and epithelial cells. This is consistent with previous research showing that IL-17 is also produced by neutrophils [30] and epithelial cells [21]. It is noteworthy that the level of IL-23 was modest with respect to IL-17, in all determinations performed. In addition, the seeming independence of IL-17 from IL-23 could be due to prompt reutilization of IL-23 by T cells. Alternatively, expansion of Th17 cells could occur, even when IL-23 levels are low, as has been described in other experimental models [31].

Halofuginone, a low molecular weight derivative of the plant alkaloid febrifugine, is able to inhibit collagen α1 gene expression in several animal models of fibrosis and cancer [32], [33], [34]. Recently, it has been reported that halofuginone inhibits transforming growth factor-β (TGF- β) signaling [33] which is known to drive differentiation of Th17 cells in the mouse. It has recently been reported that halofuginone induces selective inhibition of Th17 differentiation and does not influence Th1, Th2, or T_reg_ differentiation [25]. In our experimental system the infection was exacerbated and IL-17 secretion was inhibited by halofuginone treatment. Compelling evidence revealed a critical role for IL-17 in the induction of natural immune genes, including antimicrobial peptides such as defensins, S100, mucins, etc [35], [36], [37], [38]. In our experimental system beta defensin 1 was not recovered in the vagina, conversely a strong increase of β2 defensin and a significant increase of beta 3 defensin were manifested during the infection. Halofuginone treatment, which strongly impaired IL-17 secretion, also caused a significant decrease of beta defensin 2 and 3. The direct relationship between the presence of IL-17 and beta defensin 2 and 3 was also corroborated by the drastic increase of beta defensin 2 after treatment with rIL-17.

Th17 responses have been shown to be involved in the protective response against fungal and bacterial mucosal infections [39], [40], [41]. Current literature attributes a critical role in neutrophil recruitment to IL-17 [42]. In a mouse model of systemic candidiasis a protective role was attributed to IL-17 because of its ability to induce neutrophil recruitment [18]. The mechanism by which Th17 immunity leads to resistance to OPC involves both IL-17-induced neutrophil recruitment and direct IL-17-induced antimicrobial effects. In our system the increase of IL-17 in the vaginal lumen and its secretion by vaginal cells seems to be independent of the neutrophil influx. As a matter of fact the robust early neutrophil migration observed soon after infection (Fig. 2) seems mainly attributable to chemotactic molecules, produced by epithelial cells following interaction with *C. albicans* [38]. Indeed the level of neutrophils also remained high during the resolution of infection, while the IL-17 production paralleled the course of infection. Given that a correlation between infiltration of polymorphonuclear neutrophils and symptomatic vulvovaginal candidiasis has been observed [43], the lack of correlation between the presence of IL-17 and neutrophil infiltration suggests the role of IL-17 may be to protect from, rather than to participate in, the inflammatory response.

As previously reported, we observed a massive influx of neutrophils to the vagina [10]. These cells were unable to inhibit the growth of *C. albicans* yeast and hyphae formation, despite their primary role as anti-*Candida* effector cells. There are two possible explanations for this inefficiency in the neutrophil performance: one is that the neutrophil influx occurred when the yeast-mycelial transition had already finished, the second is that neutrophils, due to microenvironmental conditions, are unable to inhibit *Candida* growth in the vaginal compartment.

Epithelial cells could indeed strongly contribute to *Candida* clearance [44], [45] and vaginal epithelial cells are described as having an important role in retarding or arresting *C. albicans* growth in a non inflammatory manner [46].

Our results show for the first time that a robust Th17 response is found to occur in the vagina during vaginal candidiasis, and IL-17 plays a role in controlling *C. albicans* infection as it induces vaginal epithelial cells to produce antimicrobial peptides.

## Materials and Methods

### Ethics Statement

All animal experiments adhered to the EU Directive 86/609. Experiments were performed according to the guidelines of the European Convention for the Protection of Vertebrate Animals used for Experimental and other Scientific Purposes. (ETS No. 123). The protocol was approved by Perugia University Ethics Committee (Comitato Universitario di Bioetica) (permit numbers 41-2005B and 34/2003-A). All efforts were made to minimize suffering during experiments.

### Microorganisms

In this study a novel reporter system for imaging *Candida albicans* infections was used. This luciferase reporter was constructed by fusing a synthetic codon-optimized version of the *Gaussia princeps* luciferase gene to *PGA59* gene of *C. albicans* (strain CA1398), which encodes a glycosylphosphatidylinositol-linked cell wall protein, under the control of ACT1 promoter [19]. For experimental infections, cells from stock cultures in YPD agar with chloramphenicol (1% yeast extract, 2% peptone, 2% glucose, all w/v) were grown in YPD medium for 24 h, then harvested by centrifugation, washed and counted as previously described [20] in an hemocytometer, and resuspended to the desired concentration in sterile physiological saline. *Candida albicans* cells were inactivated by heating at 60°C for 30 minutes.

### Mice

Female CD1 mice obtained from Harlan Italy Laboratories (Udine, Italy) were used at seven weeks of age. Mice were allowed to rest for 1 week before the experiment; by that time the animals were roughly 8 weeks old.

### Animal model of vaginal infection

The mouse model of vaginal infection has been described previously [19], [20]. Six days prior to infection a pseudo-estrus condition was induced in mice by subcutaneous injection of 0.2 mg of estradiol valerate in 100 µl of sesame oil (Sigma-Aldrich); this was repeated weekly until the completion of the study. Mice anesthetized with 2.5 (v/v) isoflurane gas were infected twice at a 24 h interval with 10 µl of a 10^9^/ml, 5×10^8^/ml or 10^7^/ml of *C. albicans* cell suspensions administered by mechanical pipette into the vaginal lumen, close to the cervix. Following infection the fungal burden was monitored by an in vivo imaging system and by analysis of the vaginal wash (Figure 1A).

### Monitoring of mouse vaginal infection

To monitor the course of infection, at selected days post-infection (starting 4 days after the challenge), 10 µl (0.5 mg/ml in 1∶10 methanol∶H_2_0) of coelenterazine (Synchem, OHM) were added to the vaginal lumen. Afterwards, mice were imaged in the IVIS-200TM imaging system under anaesthesia using 2.5% isoflurane. Total photon emission from vaginal areas within the images of each mouse was quantified as previously described [19], [20]. The vaginal lavages were conducted using 150 µl of saline with repeated aspiration for 10 to 20 times and the fluid was serially diluted and plated on YPD agar plus chloramphenicol. CFU were then evaluated and expressed as CFU/ml.

### Vaginal cell collection and processing

The vaginal washes obtained at different days after infection were treated with protease inhibitors (Complete Protease Inhibitor Cocktail, Sigma-Aldrich) and centrifuged. After centrifugation at 600×g the supernatants were recovered and stored at −20°C and the cells of the vaginal wash were fixed with 4% of PFA. To analyze the polymorphonuclear cell number in the vaginal wash the cells were incubated with rat anti-mouse Ly6G FITC conjugate for 45 minutes on ice. Labelled cells were analyzed by a cytofluorimeter. The cells were first analyzed using FSC/SSC parameter and polymorphonuclear cells were gated according to their size and granularity, then gated cells were tested for Ly6G expression.

After vaginal infection, at different time points, the vaginas were aseptically removed from dead mice; the vaginal tissue was cut longitudinally and minced with sterile scalpel in cRPMI medium consisting of RPMI 1640 supplemented with 10% heat-inactivated fetal calf serum and 100 µl/ml penicillin streptomycin (Gibco).The tissues were digested with cRPMI supplemented with sterile 0.25% collagenase from *Clostridium histolyticum* (Sigma-Aldrich), following incubation for 30 minutes at 37°C [47]. After digestion, tissues and cells were filtered with a cell strainer 100 µm (BD Falcon) and washed with RPMI 1640 medium. To evaluate the cytokine production, the vaginal cells were counted by a hemocytometer, left untreated or re-stimulated with 4×10^6^ heat inactivated *C. albicans*, and incubated for 72 hours in cRPMI at 37°C. The supernatants from the culture wells were treated with protease inhibitors and stored at −20°C until the ELISA test, and the vaginal cells were fixed with 4% of PFA.

### Lymph node collection and processing

Vaginal draining lumbar lymph nodes were identified on the posterior abdominal wall lateral to the inferior vena cava and abdominal aorta, respectively. These lymph nodes were excised in cRPMI medium, homogenized and counted with a hemocytometer. To evaluate the cytokine production, the lymph node cells (2×10^6^/ml) were left untreated or re-stimulated with 4×10^6^ heat inactivated *C. albicans* for 72 hours in cRPMI at 37°C. The supernatants from the culture wells were treated with protease inhibitors and stored at −20°C until the ELISA test. The lymph nodes cells were fixed with 4% of PFA.

### Detection of intracellular and supernatant cytokines and flow cytometry

The supernatants of vaginal washes, and vagina and lymph node cell cultures, obtained from infected and non-infected mice were assayed for the presence of IL-17 and IL-23 cytokines using the enzyme-linked immunosorbent ELISA cytoset (eBioscience and Biosource respectively). The analysis of surface molecules, intracellular IL-17 and β-defensin contents of vaginal and lymph node cells was performed by flow cytometry using standard methodology for direct and indirect immunofluorescence. Briefly, 2×10^5^ cells treated with 10 µg/ml of Brefeldin A (Calbiochem) for 3 h, were incubated with PBS-S buffer (PBS with 0.1% of saponin) for 45 minutes at 4°C with combinations of different antibodies. For IL-17 detection, cells were labelled with rat anti mouse IL-17 PE conjugate (BD Pharmingen) and either rat anti mouse CD4-FITC conjugate (Santa Cruz Biotechnology) or rat anti mouse Gr-1 FITC conjugate; for β-defensin detection, rabbit anti mouse β-defensin 1, goat anti mouse β-defensin 2 or goat anti mouse - β defensin 3 (Santa Cruz Biotechnology), with goat anti-rabbit TRIC conjugate or rabbit anti goat PE conjugate (Sigma-Aldrich), were used. For epithelial cell labelling, a FITC conjugate antibody anti-pan cytokeratin (Sigma) was used. Stained cells were washed with PBS-S buffer, resuspended in fluorescent buffer (PBS with 1% FBS and 0.5% NaN_3_) and analyzed using a FACScan cytofluorimeter (Becton Dickinson, BD). The acquired data were analyzed with CELLQuest software (BD).

### Histological analysis

For histological evaluation, the mice were sacrificed, and the vaginas were removed and immediately fixed in 10% (v/v) neutral buffered formalin for 24 h. They were then dehydrated, embedded in paraffin, sectioned into 3- to 4-µm-thick sections, and stained with periodic acid-Schiff reagent.

### Microscopic analysis of vaginal wash cells

The vaginal wash cells were cytospun onto a glass slide (400×g for 7 min) and air dried for 1 h. Cells were then stained using Diff-Quick staining and examined under a light microscope at a magnification of ×40. Neutrophils were identified by their characteristic tri-lobar nucleus.

### Halofuginone treatment

Mice were injected intraperitoneally with 5 µg/100 µl or 10 µg/100 µl of a solution of halofuginone (obtained from Collgard Biopharmaceuticals (Tel Aviv, Israel) or diluent, two days before and every two days after infection. In parallel experiments and at the same time as the halofuginone treatment, mice were treated intravaginally with 10 µl (10 pg/mouse) of recombinant mouse IL-17 (eBioscience).

### Statistical analysis

Photon Flux emission, CFU counts, PMN count, IL-17, IL-23 and beta-defensin production were compared using the non-parametric Mann–Whitney U-test. The alpha value was set at 0.05. The correlation between the Total Photons emitted and CFU count in the vaginal wash was assessed with the Pearson's correlation test.

## References

[1] Fidel PL, Sobel JD (1996). Immunopathogenesis of recurrent vulvovaginal candidiasis.. Clin Microbiol Rev.

[2] Sobel JD (2007). Vulvovaginal candidosis.. Lancet.

[3] Magliani W, Conti S, Cassone A, De Bernardis F, Polonelli L (2002). New immunotherapeutic strategies to control vaginal candidiasis.. Trends Mol Med.

[4] Fidel PL (2004). History and new insights into host defense against vaginal candidiasis.. Trends Microbiol.

[5] Cassone A, De Bernardis F, Santoni G (2007). Anticandidal immunity and vaginitis: novel opportunities for immune intervention.. Infect Immun.

[6] Naglik JR, Fidel PL, Odds FC (2008). Animal models of mucosal Candida infection.. FEMS Microbiol Lett.

[7] Fidel PL (2007). History and update on host defense against vaginal candidiasis.. Am J Reprod Immunol.

[8] Fidel PL, Wolf NA, KuKuruga MA (1996). T lymphocytes in the murine vaginal mucosa are phenotypically distinct from those in the periphery.. Infect Immun.

[9] Nandi D, Allison JP (1991). Phenotypic analysis and gamma delta-T cell receptor repertoire of murine T cells associated with the vaginal epithelium.. J Immunol.

[10] Fidel PL, Luo W, Steele C, Chabain J, Baker M (1999). Analysis of vaginal cell populations during experimental vaginal candidiasis.. Infect Immun.

[11] Taylor BN, Saavedra M, Fidel PL (2000). Local Th1/Th2 cytokine production during experimental vaginal candidiasis: potential importance of transforming growth factor-beta.. Med Mycol.

[12] Ibraghimov AR, Sacco RE, Sandor M, Iakoubov LZ, Lynch RG (1995). Resident CD4+ alpha beta T cells of the murine female genital tract: a phenotypically distinct T cell lineage that rapidly proliferates in response to systemic T cell activation stimuli.. Int Immunol.

[13] Saavedra M, Taylor B, Lukacs N, Fidel PL (1999). Local production of chemokines during experimental vaginal candidiasis.. Infect Immun.

[14] Harrington LE, Hatton RD, Mangan PR, Turner H, Murphy TL (2005). Interleukin 17-producing CD4+ effector T cells develop via a lineage distinct from the T helper type 1 and 2 lineages.. Nat Immunol.

[15] Matsuzaki G, Umemura M (2007). Interleukin-17 as an effector molecule of innate and acquired immunity against infections.. Microbiol Immunol.

[16] Huang W, Na L, Fidel PL, Schwarzenberger P (2004). Requirement of interleukin-17A for systemic anti-Candida albicans host defense in mice.. J Infect Dis.

[17] Conti HR, Shen F, Nayyar N, Stocum E, Sun JN (2009). Th17 cells and IL-17 receptor signaling are essential for mucosal host defense against oral candidiasis.. J Exp Med.

[18] Pirofski LA, Casadevall A (2009). Rethinking T cell immunity in oropharyngeal candidiasis.. J Exp Med.

[19] Enjalbert B, Rachini A, Vediyappan G, Pietrella D, Spaccapelo R (2009). A multifunctional, synthetic Gaussia princeps luciferase reporter for live imaging of Candida albicans infections.. Infect Immun.

[20] Pietrella D, Rachini A, Torosantucci A, Chiani P, Brown AJ (2010). A beta-glucan-conjugate vaccine and anti-beta-glucan antibodies are effective against murine vaginal candidiasis as assessed by a novel in vivo imaging technique.. Vaccine.

[21] Cua DJ, Tato CM (2010). Innate IL-17-producing cells: the sentinels of the immune system.. Nat Rev Immunol.

[22] Acosta-Rodriguez EV, Rivino L, Geginat J, Jarrossay D, Gattorno M (2007). Surface phenotype and antigenic specificity of human interleukin 17-producing T helper memory cells.. Nat Immunol.

[23] Iwakura Y, Ishigame H (2006). The IL-23/IL-17 axis in inflammation.. J Clin Invest.

[24] Bettelli E, Korn T, Oukka M, Kuchroo VK (2008). Induction and effector functions of T(H)17 cells.. Nature.

[25] Sundrud MS, Koralov SB, Feuerer M, Calado DP, Kozhaya AE (2009). Halofuginone inhibits TH17 cell differentiation by activating the amino acid starvation response.. Science.

[26] Ganz T (1999). Defensins and host defense.. Science.

[27] Ganz T (2002). Immunology. Versatile defensins.. Science.

[28] Liang SC, Tan XY, Luxenberg DP, Karim R, Dunussi-Joannopoulos K (2006). Interleukin (IL)-22 and IL-17 are coexpressed by Th17 cells and cooperatively enhance expression of antimicrobial peptides.. J Exp Med.

[29] Ildgruben AK, Sjoberg IM, Hammarstrom ML (2003). Influence of hormonal contraceptives on the immune cells and thickness of human vaginal epithelium.. Obstet Gynecol.

[30] Ferretti S, Bonneau O, Dubois GR, Jones CE, Trifilieff A (2003). IL-17, produced by lymphocytes and neutrophils, is necessary for lipopolysaccharide-induced airway neutrophilia: IL-15 as a possible trigger.. J Immunol.

[31] Miyahara Y, Odunsi K, Chen W, Peng G, Matsuzaki J (2008). Generation and regulation of human CD4+ IL-17-producing T cells in ovarian cancer.. Proc Natl Acad Sci U S A.

[32] Elkin M, Miao HQ, Nagler A, Aingorn E, Reich R (2000). Halofuginone: a potent inhibitor of critical steps in angiogenesis progression.. FASEB J.

[33] Pines M (2008). Targeting TGFβ signaling to inhibit fibroblasts activation as a therapy for fibrosis and cancer.. Expert Opin Drug Discov.

[34] Pines M, Vlodavsky I, Nagler A (2000). Halofuginone: from veterinary use to human therapy.. Drug Develop Res.

[35] Fantini MC, Monteleone G, Macdonald TT (2007). New players in the cytokine orchestra of inflammatory bowel disease.. Inflamm Bowel Dis.

[36] Raffatellu M, Santos RL, Verhoeven DE, George MD, Wilson RP (2008). Simian immunodeficiency virus-induced mucosal interleukin-17 deficiency promotes Salmonella dissemination from the gut.. Nat Med.

[37] Shen F, Gaffen SL (2008). Structure-function relationships in the IL-17 receptor: implications for signal transduction and therapy.. Cytokine.

[38] Yano J, Lilly E, Barousse M, Fidel PL (2010). Epithelial cell-derived S100 calcium-binding proteins as key mediators in the hallmark acute neutrophil response during Candida vaginitis.. Infect Immun.

[39] Happel KI, Zheng M, Young E, Quinton LJ, Lockhart E (2003). Cutting edge: roles of Toll-like receptor 4 and IL-23 in IL-17 expression in response to Klebsiella pneumoniae infection.. J Immunol.

[40] Godinez I, Haneda T, Raffatellu M, George MD, Paixao TA (2008). T cells help to amplify inflammatory responses induced by Salmonella enterica serotype Typhimurium in the intestinal mucosa.. Infect Immun.

[41] Levitz SM (2009). Th17 cells bounce off the fungal wall.. Cell Host Microbe.

[42] Aujla SJ, Dubin PJ, Kolls JK (2007). Th17 cells and mucosal host defense.. Semin Immunol.

[43] Fidel PL (2005). Immunity in vaginal candidiasis.. Curr Opin Infect Dis.

[44] Gupta SM, Aranha CC, Mohanty MC, Reddy KV (2008). Toll-like receptors and cytokines as surrogate biomarkers for evaluating vaginal immune response following microbicide administration.. Mediators Inflamm.

[45] Han JH, Kim MS, Lee MY, Kim TH, Lee MK (2010). Modulation of human beta-defensin-2 expression by 17beta-estradiol and progesterone in vaginal epithelial cells.. Cytokine.

[46] Nomanbhoy F, Steele C, Yano J, Fidel PL (2002). Vaginal and oral epithelial cell anti-Candida activity.. Infect Immun.

[47] De Bernardis F, Lucciarini R, Boccanera M, Amantini C, Arancia S (2006). Phenotypic and functional characterization of vaginal dendritic cells in a rat model of Candida albicans vaginitis.. Infect Immun.

